# Retinoblastoma and mosaic 13q deletion: a case report

**DOI:** 10.1186/s40942-021-00321-9

**Published:** 2021-09-03

**Authors:** Pablo Gargallo, Silvestre Oltra, Julia Balaguer, Honorio Barranco, Yania Yáñez, Vanessa Segura, Antonio Juan-Ribelles, Inés Calabria, Margarita Llavador, Victoria Castel, Adela Cañete

**Affiliations:** 1 Clinical and Translational Research in Cancer, Health Research Institute La Fe (IIS La Fe), Valencia, Spain; 2 Oncology Department, Imegen-Health in Code Group, Valencia, Spain; 3 Genetics Unit, La Fe Hospital, Valencia, Spain; 4 Genetics Department, Valencia University, Valencia, Spain; 5 Pediatric Oncology and Hematology Unit, University Hospital La Fe, Valencia, Spain; 6 Pediatric Ophthalmology Unit, University Hospital La Fe Hospital, Valencia, Spain; 7 Pathology Department, University La Fe Hospital, Valencia, Spain

**Keywords:** Retinoblastoma, 13q-syndrome, Mosaicism, Cytogenetics, Molecular genetics

## Abstract

**Background:**

Patients with 13q-syndrome are at risk of retinoblastoma when the *RB1* gene, located in the chromosomal band 13q14.2, is deleted. This syndrome is frequently associated with congenital malformations and developmental delay, although these signs could be mild. Mosaic 13q-deletion patients have been previously reported in the literature; their phenotype is variable, and they may not be recognized.

**Case presentation:**

Retinoblastoma diagnosed in a child with 13q-mosaicism confirmed in blood, oral mucosa, healthy retina and retinoblastoma. A second *RB1* hit is present exclusively in the retinoblastoma sample (*RB1* c.958C>T p.Arg320Ter). Other detected molecular events in retinoblastoma are 6p12.3pter gain and 6q25.3qter loss. Clinical examination is unremarkable except for clinodactyly of the right fifth finger.

**Discussion and conclusions:**

We describe a case of mosaic 13q deletion syndrome affected by retinoblastoma. Molecular data obtained from the tumor analysis are similar to previous data available about this malignancy. High clinical suspicion is essential for an adequate diagnosis of mosaic cases.

## Background

Retinoblastoma is a rare tumor that occurs in young children’s retina. About 40% of patients diagnosed with retinoblastoma have a predisposing genetic condition [1]. Most of them carry heterozygous truncating *RB1* mutations in the germline. Some patients present isolated deletions of one of the two *RB1* alleles, and at-risk patients are exceptionally 13q-syndrome cases [2]. Because of the fact that 98% of retinoblastoma cases begin after a double *RB1* hit, according to Knudson’s hypothesis [3], all these children are at a major risk of being affected.

13q deletion syndrome was first described by Allderdice et al. after studying two pediatric patients in 1969 [4]. The first patient affected by the syndrome including retinoblastoma was reported in 1983 [5]. Several cases have been communicated during the past 50 years and the syndromic phenotype has been characterized. Intellectual disability, facial anomalies, several malformations and retinoblastoma risk stand out as the most prominent signs amongst other previously described abnormalities. However, the tumor would not be able to progress easily in 13q-syndrome even if a second *RB1* hit were present. It has been hypothesized that some genes deleted together with *RB1* would be necessary for retinoblastoma development. Available data suggest that 13q deletions larger than 1 Mb—and particularly those including *MED4* and *SUCLA2*—are associated with unilateral forms or without retinoblastoma development [6].

Improvements in cytogenetic analysis has enabled better molecular characterization of 13q-syndrome cases and more accurate genotype–phenotype correlations. Depending on the deleted chromosomal bands, three clinical groups may be established [7]:

- Group 1: 13q12.2–13q32. Mild intellectual disability, growth delay, limb malformations, and retinoblastoma risk (when the *RB1* gene is deleted [chromosomal position 13q14.2].
- Group 2: 13q32. Severe brain malformations and developmental delay.
- Group 3: 13q33–13q34. Minor congenital malformations but severe intellectual impairment.

Some patients with 13q-syndrome are affected by a mosaic disease and a few cases have been described [8–11]. Bestetti et al. reported a patient with mosaic 13q deletion syndrome including *RB1* but no retinoblastoma [8].

### Case presentation

A 6-month-old girl conceived by in vitro fertilization (IVF) (own oocytes and anonymous donor sperm) was admitted to the hospital because of leukocoria and strabismus. Past medical history and physical examination were unremarkable except for clinodactyly of the right fifth finger. Indirect ophthalmoscopic examination and examination under anesthesia was performed by ophthalmologists. Orbital ultrasound and magnetic resonance imaging (MRI) scans showed a 14 × 13 × 11 mm left intraocular mass located in the lower-external retinal side. Retinal detachment was also detected. Other tumoral lesions were ruled out by an ophthalmologist and MRI in both retina and brain. Diagnosis of Retinoblastoma was made and, based on *International Classification for Intraocular Retinoblastoma,* a grade E was established. The patient received intra-arterial melphalan but due to a local vasospasm in her left leg, the treatment was discontinued. Afterwards, four courses of conventional chemotherapy were administered (vincristine, carboplatin and etoposide). A partial response was achieved, but, despite chemotherapy, the disease progressed few weeks later and the affected eye was enucleated.

**Fig. 1:**
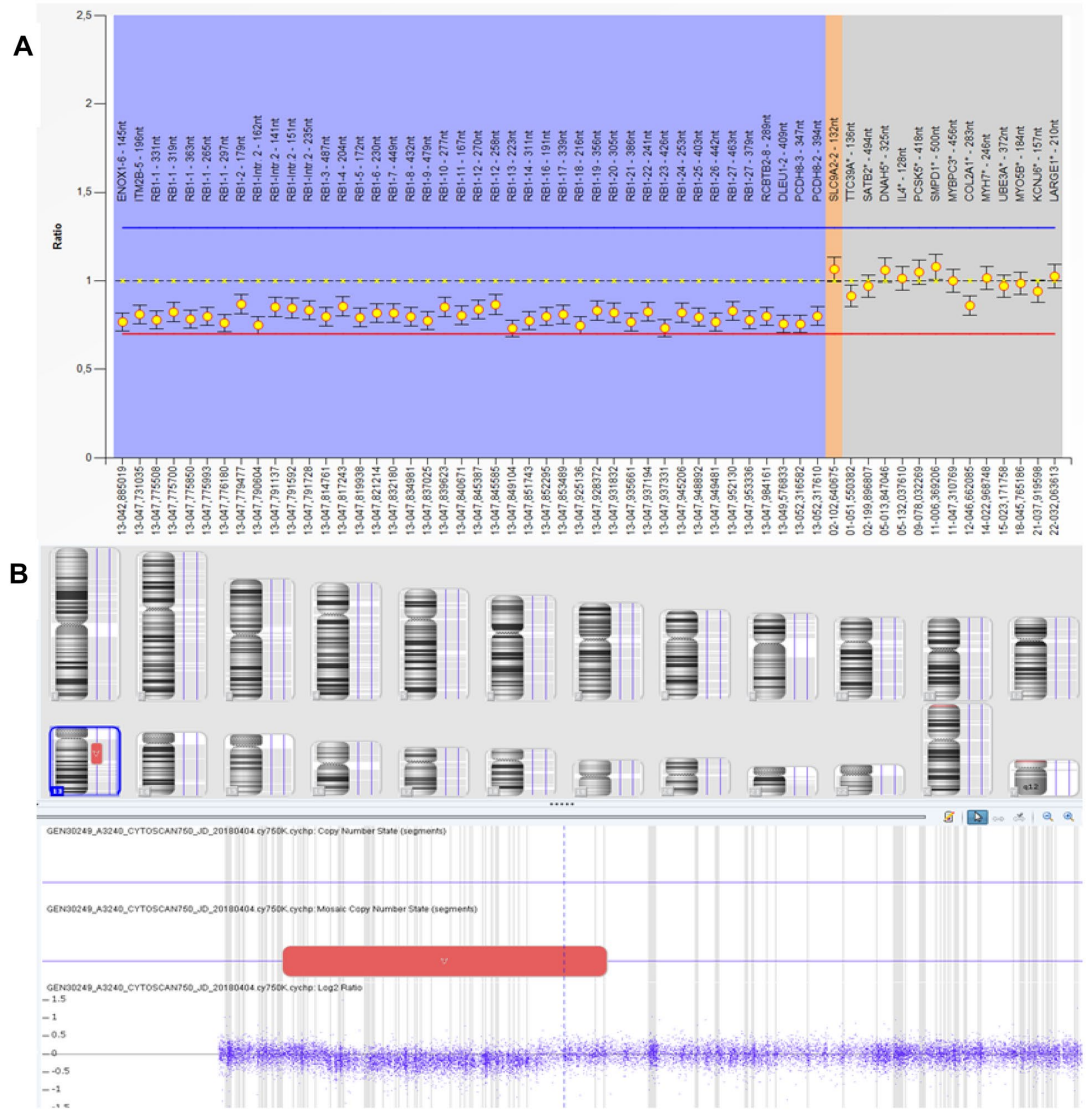
*RB1* deletion in the context of mosaic 13q deletion. **A** Multiplex Ligation-dependent Probe Amplification (MLPA) assay looking for *RB1* gene deletions or duplications (SALSA P047-C1) in germline DNA from peripheral blood lymphocytes. The detected values were low but not consistent with a heterozygous deletion. The suspicion was a complete *RB1* deletion in mosaicism. **B** Genomic SNP array (AffymetrixCytoScan 750 array) reports a 13q deletion in mosaicism. It is a deletion of 35.7 Mb from 13q12.13 to 13q21.2 (arr[hg19] 13q12.13q21.2(26,555,387–62,280,955) × 1–2) observed in about 40% of all determinations

**Fig. 2:**
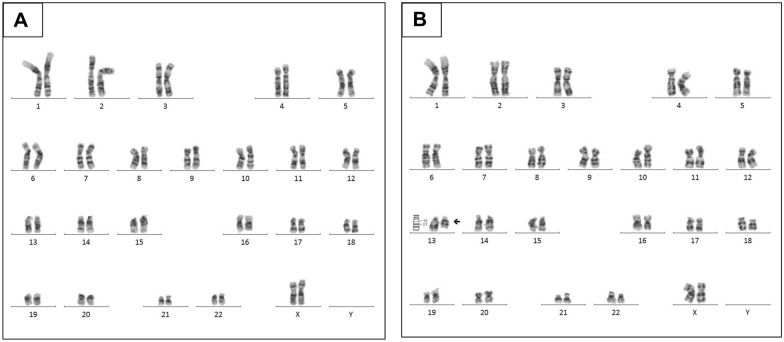
Cytogenetic karyotype from cultivated lymphocytes previously stimulated with phytohemagglutinin. Karyotype 46,XX,del(13)(q12q21)[6]/46,XX[44]. **A** A majority cell line (44 cells): 46 chromosomes whose identification with G bands (resolution level of 400–500 bands) does not show numerical or structural alterations (46, XX). **B** A minor cell line (6 cells): 46 chromosomes but shows the presence of an interstitial deletion in the long arm of chromosome 13

On the basis of global recommendations, the *RB1* gene was studied in germline DNA from peripheral blood lymphocytes. Exon–intron boundaries of *RB1* were amplified by conventional PCR and then sequenced by the Sanger method; no mutations were detected. A Multiplex Ligation-dependent Probe Amplification (MLPA) assay was used to test for *RB1*-gene deletions and duplications (SALSA P047-C1). The detected values were relatively low but within the normal range (Fig. 1A) and a complete *RB1* deletion in mosaicism was suspected. A genomic SNP array (AffymetrixCytoScan 750 array) was performed and a 13q deletion of 35.7 Mb from 13q12.13 to 13q21.2 (arr[hg19] 13q12.13q21.2(26,555,387–62,280,955) × 1–2) detected in around 40% of cells (Fig. 1B) was confirmed. This result was further confirmed by cytogenetic karyotype analysis of cultivated lymphocytes previously stimulated with phytohemagglutinin. Fifty metaphases were analyzed and two cell clones were detected. A majority cell line (44 cells) presented 46 chromosomes whose identification with G bands (resolution level of 400–500 bands) did not show numerical or structural alterations (46, XX). A minor cell line (6 cells) with 46 chromosomes showed the presence of an interstitial deletion in the long arm of chromosome 13 (Fig. 2). A *RB1*-specific FISH probe (LSI13) performed from swab oral mucosa cells evidenced 13q deletion in around 40% of the cells.

**Fig. 3:**
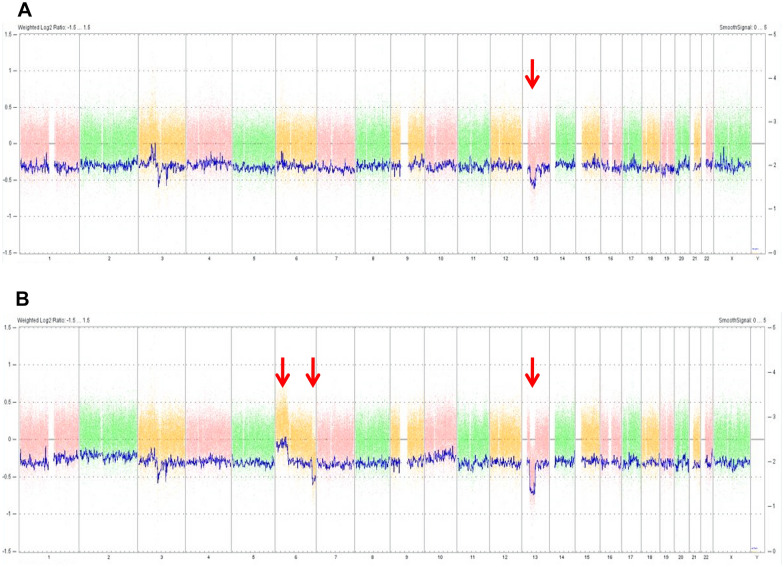
Affymetrix Oncoscan array performed from tumor-free paraffin-embedded retina and also from fixed retinoblastoma sample. The results have been analyzed with the Chromosome Analysis Suite software, applying the following filters in the analysis: at least 500 altered markers at 500 kb for CNV and at least 1 marker altered in 20 mb for LOH. The genome version was Hg19. Oncoscan by Affymetrix does not allow calculating the mosaicism percentage; therefore, the figures obtained are an approximation. **A** Affymetrix Oncoscan array from tumor-free paraffin-embedded healthy retina. It carries the 13q deletion in mosaicism but in about 50% of studied cells. **B** Affymetrix Oncoscan array from fixed retinoblastoma sample. 13q deletion is detected with a frequency consistent with heterozygosity in all tumor cells. Neither LOH nor chromothripsis in 13q bands were detected. 6p12.3pter gain and 6q25.3qter loss were detected as well

We performed an Affymetrix Oncoscan array for both her tumor-free paraffin-embedded retina and fixed retinoblastoma sample. The healthy retina carried the 13q deletion in mosaicism but in about 50% of the studied cells. However, all retinoblastoma sample cells carried the deletion in heterozygosity (Fig. 3). Neither LOH (Loss of Heterozygosity) nor chromothripsis were detected in 13q bands. Furthermore, 6p12.3pter gain (3 total copies) and 6q25.3qter loss (1 total copy) were reported exclusively in the tumor sample.

Looking for second hit mutations in *RB1,* we applied a custom designed NGS panel (*Onconano V2*) that included the *RB1*, *BCOR* and *CREBPP* genes (among other 400 commonly mutated genes in pediatric cancer). The study detected only one pathogenic single-nucleotide variant, *RB1* c.958C>T (p.Arg320Ter) (NM_000321.2 chromosomal position 13–48,941,648-C-T; allele frequency of 25%). Copy number variations in 6p, 6q and 13q were again observed.

**Fig. 4:**
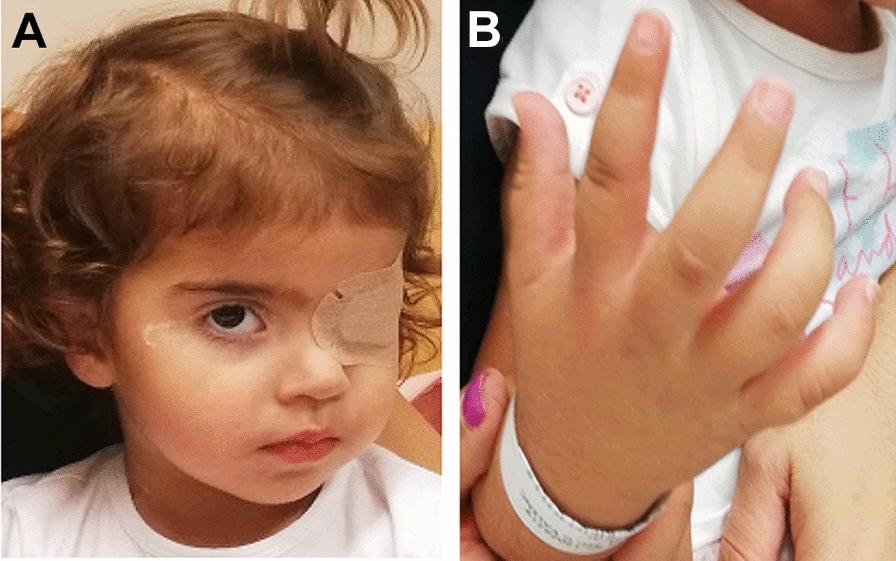
**A** Patient’s face. Left eye enucleated and waiting for prosthetics at the time of taking the photo. The patient’s face has no noteworthy malformations. The wavy hair is not striking, the length of the forehead does not seem pathological at the age of 2. Other facial features are considered normal. **B** Right hand. Clinodactyly of the right fifth finger. She has no other limb malformations

After molecular diagnosis and completing the treatment, the patient was placed on surveillance. The right eye has been free of disease and the child is 42 months old now. She does not present growth retardation at the moment (weight and height in the 50th percentile; cranial perimeter in the 90th). Neither cardiac, eye nor other malformations have been detected and neurological development has been normal (Fig. 4).

Informed consent for genetic studies and for taking and sharing pictures was obtained from both parents.

## Discussion and conclusions

We described the case of a child with 13q-mosaicism affected by retinoblastoma. The unilateral presentation agrees with previous data available for 13q deletions larger than 1 Mb including *MED4* and *SUCLA2* [6]. As in this case, retinoblastoma with both genes deleted is associated with less tumor aggressiveness compared with tumors whose genes are conserved [6].

Retinoblastoma seems to be caused by a double hit in *RB1* approaching 98% cases (by mutation, deletion, promoter methylation or intra-genic chromothripsis) [12, 13]. Few retinoblastoma cases would start because of *MYCN* amplification [13]. 13q deletion syndrome patients would not be an exception. In fact, we confirmed a second *RB1* hit (*RB1* p.Arg320Ter) in the tumor.

However, double *RB1* hit only gives rise to retinoma; therefore, subsequent epigenetic or genetic changes would give an advantage for tumor progression. The sequence of events capable of causing a malignant phenotype is only partially known. Epigenetic deregulation secondary to homozygous *RB1* loss drives an increase in KIF14 and E2F3 levels [14] and could lead to the expression of the *SYK* oncogene as well. Moreover, cellular control mediated by p53 is inactivated as a result of high expression of *MDM2* and *MDM4* in retinoblastoma [14].

In addition, cytogenetic analysis has shown recurrent CNVs (copy number variation) among retinoblastoma tumors, which are mainly chromosomal gains at 1q, 2p, 6p, 13q and 19q and losses at 13q, 16q and 17p [15]. These recurrent aberrations allow to establish as a possible hypothesis that genes located at these loci could be related to retinoblastoma progression [15], yet no conclusive data are available about this at the moment. We looked for CNVs in the tumor and discovered a chromosomal gain in 6p12.3pter, which is one of the most frequently reported CNVs in retinoblastoma [15]. However, we also detected a less common deletion of 6q25.3qter. The deletion of this region has already been described among non-13q-deletion syndrome patients, although rarely [16]. Sixty OMIM genes are located in this region, and several of them are associated with different cancers, but none with retinoblastoma. A terminal 6q deletion may be present in ovarian cancer and neuroblastoma [17] and seems to be related to bad prognosis in neuroblastoma [17]. The fact that this deletion could play a role in retinoblastoma development in the context of 13q-syndrome is unknown.

Furthermore, NGS approaches have detected a low rate of mutations in retinoblastoma. Several studies support retinoblastoma as one of the less mutated human tumors. Only *BCOR* (mutated in 13% of tumors) and *CREBPP* mutations occur frequently in retinoblastoma [18]. Therefore, retinoblastoma presents a stable genome with few genetic events described and epigenetic deregulation appears to have a notable role [19]. Studies based on RNA-sequencing could continue to shed light on the genes and signaling pathways involved in retinoblastoma development [20]. In regards to common mutated genes in retinoblastoma, we determined *BCOR* and *CREBPP* status without detecting pathogenic variants. We did not find other variants considered pathogenic or likely pathogenic in 400 genes commonly mutated in pediatric cancer beyond *RB1*.

The patient carries the deletion 13q12.13–13q21.2 and, therefore, fits in Group 1 of the clinical classification for 13q-syndrome [7]. Patients with band 13q14 deleted typically present with mild facial anomalies such as high forehead, short nose, small upper lip, curly hair and down-turned corners of the mouth [6]. Our patient does not show these facial features. Furthermore, deletion of *NUFIP1,* located in 13q14.12, and *PCDH8,* in 13q21.1, may be crucial for developmental delay [6]. Both of them are deleted in our patient, but the degree of mosaicism in her central nervous system is unknown. In fact, she is neurologically normal. Moreover, other common abnormalities in Group 1 are micrognathia and microcephaly but these are related to loss in the 13q21.33q31.1 and 13q21.32q21.33 regions, respectively [6]. Our patient’s deletion finishes at 13q21.2; therefore, she does not present either micrognathia or microcephaly, because those regions are not affected. About 75% of patients with large deletions present short height, but this is not the case of our patient (50th percentile). Genes involved in short height have not been clearly defined.

The *BRCA2* gene, located in 13q13.1, may be lost in some 13q-patients. Heterozygous mutations in this gene predispose to breast and ovarian cancer syndrome in adulthood [21] and a complete deletion of this gene might predispose to these tumors as well. However, the occurrence of these two tumors has not been reported in 13q-syndrome to date. Our patient loses *BRCA2;* therefore, she may benefit from risk-adapted surveillance strategies for breast/ovarian cancer.

After confirming retinoblastoma diagnosis in a child, genetic study of *RB1* in the germline is mandatory. Any phenotypic manifestation, including minor peculiarities (clinodactyly of the fifth finger in our case) should raise suspicion of 13q-syndrome, and it should be studied, given the fact that mosaic forms exist.

## Data Availability

Data generated or analyzed during this study are included in this published article.

## Abbreviations

MRI: Magnetic resonance imaging
LOH: Loss of heterozygosity
CNV: Copy number variation
NGS: Next generation sequencing
